# Communicator Extraordinaire: Extracellular Vesicles in the Tumor Microenvironment Are Essential Local and Long-Distance Mediators of Cancer Metastasis

**DOI:** 10.3390/biomedicines11092534

**Published:** 2023-09-14

**Authors:** Megan I. Mitchell, Olivier Loudig

**Affiliations:** Center for Discovery and Innovation, Hackensack Meridian Health, Nutley, NJ 07110, USA; megan.mitchell@hmh-cdi.org

**Keywords:** extracellular vesicles, cancer, metastasis, biomarkers, cell-to-cell communication

## Abstract

Human tumors are increasingly being described as a complex “ecosystem”, that includes many different cell types, secreted growth factors, extracellular matrix (ECM) components, and microvessels, that altogether create the tumor microenvironment (TME). Within the TME, epithelial cancer cells control the function of surrounding stromal cells and the non-cellular ECM components in an intricate orchestra of signaling networks specifically designed for cancer cells to exploit surrounding cells for their own benefit. Tumor-derived extracellular vesicles (EVs) released into the tumor microenvironment are essential mediators in the reprogramming of surrounding stromal cells, which include cancer-associated fibroblasts (CAFs), tumor-associated macrophages (TAMs), tumor-infiltrating lymphocytes (TILs), and tumor endothelial cells (TECs), which are responsible for the promotion of neo-angiogenesis, immune cell evasion, and invasion which are essential for cancer progression. Perhaps most importantly, tumor-derived EVs play critical roles in the metastatic dissemination of tumor cells through their two-fold role in initiating cancer cell invasion and the establishment of the pre-metastatic niche, both of which are vital for tumor cell migration, homing, and colonization at secondary tumor sites. This review discusses extracellular vesicle trafficking within the tumor microenvironment and pre-metastatic niche formation, focusing on the complex role that EVs play in orchestrating cancer-to-stromal cell communication in order to promote the metastatic dissemination of cancer cells.

## 1. Introduction

Although solid tumors are generally composed of monoclonal cancerous epithelial cells, they are a complex “ecosystem” that incorporates different cell types, secreted factors, extracellular matrix (ECM), and microvessels, all contributing to this biological entity [1]. The infiltration of stromal cells such as inflammatory immune cells (i.e., macrophages, dendritic cells, neutrophils, and myeloid-derived suppressor cells), adipocytes, fibroblasts, and endothelial cells in addition to non-cancerous and cancerous epithelial cells, all contribute to what is termed the tumor microenvironment (TME) [2,3,4]. The TME comprises cancerous epithelial cells that have gained the ability to control the function of surrounding stromal cells and the secretion/composition of non-cellular ECM components in an intricate orchestra of signaling networks designed for these cancer cells to exploit surrounding non-malignant cells to their benefit [5]. The “reverse Warburg effect” is one classic example wherein epithelial cancer cells induce the metabolic reprogramming of surrounding stromal cells to enhance aerobic glycolysis and their production of lactate [6]. Cancer cell metabolism is unique when compared to that of normal cells in that even in the presence of sufficient oxygen, tumor cells rely predominantly on oxidative phosphorylation (OXPHOS) rather than glycolysis for the production of adenosine triphosphate (ATP) (i.e., energy) [7,8]. This process was described in 1927 by Otto Warburg and has been termed the “Warburg effect” [9]. In addition to the enhanced energy supply that the recruitment of stromal cells into the TME has, the infiltration of stromal cells has also extensively been shown to promote neo-angiogenesis [10], epithelial cell migration [11,12], and extracellular matrix remodeling [13,14]. Additionally, stromal influences are known to contribute significantly to both the evasion of immune surveillance and enhanced chemotherapeutic resistance of cancer cells [15,16], which are largely attributed to the increased production of several stromal derived chemokines, cytokines [17,18], that include tumor necrosis factor alpha (TNF-α) [19], interleukin-6 (IL-6) [20], transforming growth factor beta (TGF-β) [21], and interleukin-10 (IL-10) [22], and growth factors. In recent years, however, circulating extracellular vesicles and other types of extracellular vesicles have gained in importance with regards to the delivery and biological effects of their packaged cargo [23,24,25]. Indeed, these membrane-bound biological nanoparticles have been shown to deliver a variety of important signals locally as well as at long distances in the organism to enable cell-to-cell communication. To date, the known ways of cellular communication that have been clearly identified for extracellular vesicles during metastasis include (1) their transfer from one cancer cell to another, to promote epithelial-to-mesenchymal transition (EMT) and regulate metastasis, and (2) their transfer from cancer cells to stromal cells and vice versa [26,27,28].

**Figure 1:**
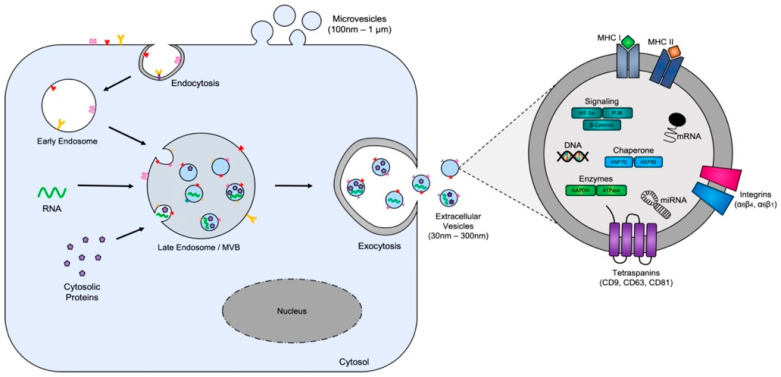
Extracellular vesicle production. Extracellular vesicles, and in particular exosomes, originate from multivesicular bodies (MVBs) which are formed during the inward budding of the plasma membrane. The subsequent inward budding of the MVB membrane encapsulates and packages cytosolic components resulting in the formation of EVs. EV cargo comprises selectively packaged proteins (e.g., tetraspanins, cytoplasmic proteins, and enzymes), nucleic acids (e.g., DNA, RNA, and miRNAs) and lipids, which have been shown to be cell-type dependent reflecting the metabolic status of their cells of origin. The subsequent fusion of MVBs with the plasma membrane results in the release of EVs into the tumor microenvironment.

Extracellular vesicles (EVs), a term which is collectively used to describe nanosomes, exosomes, microvesicles (MVs), and apoptotic bodies, are small bi-layered membrane-bound vesicles, typically ranging from 8 to 12 nm, 30 to 150 nm, 200 to 1000 nm, and 500+ nm in diameter, respectively [29]. Apoptotic bodies are formed during the process of apoptotic cell death when the cytoskeleton of cells breaks down resulting in the outward blebbing of the membrane and the splitting of cellular content into distinct membrane-enclosed vesicles [30]. Microvesicles and nanovesicles are formed via the outward blebbing and detachment of the plasma membrane. While not completely understood, it is believed that the mechanism governing the formation and release of microvesicles is a controlled process which utilizes the endosomal machinery for vesicle formation [31]. Exosome formation, which is distinct from the formation of both apoptotic bodies and microvesicles, originates from the inward budding of the plasma membrane with the subsequent formation of multivesicular bodies (MVBs) through the endosomal pathway which are ultimately released into the extracellular matrix upon the fusion of MVBs with the plasma membrane [32] (Figure 1). The international society for extracellular vesicles (ISEV) published the minimal information for studies of extracellular vesicles 2018 (MISEV2018) guidelines wherein they endorse the utilization of the term “extracellular vesicle” (EV) for any naturally released particles from cells which are enclosed by a double lipid membrane and cannot self-replicate. This is because the assignment of EVs to a particular biogenesis pathway remains extraordinarily difficult; as such, the ISEV urges authors to make use of the nomenclature “EVs” in place of terms such as “exosomes” and “microvesicles” [33]. In accordance with these guidelines in this review we utilize the term EV for all descriptions; however, where appropriate if cited papers have utilize the term “exosome” and the manuscript contains sufficient data that include multiple experimentation methods demonstrating biophysical characteristics of EVs (i.e., including the composition of tetraspanin markers, transmission electron microscopy, and/or nanoparticle tracking), and descriptions of cellular origin, we refer to these vesicles as “exosome-like vesicles” in this manuscript. Exosome-like vesicles are known to carry common surface molecules that include several tetraspanin transmembrane proteins such as Alix, CD63, CD81, and CD9 [34,35,36], but EVs in general can also carry these plasma membrane markers in addition to other cell-type specific surface markers that are acquired during their normal biogenesis. Particular examples include: prostate specific antigen (PSA) for exosome-like vesicles produced by prostate cells [37], asialoglycoprotein receptor 1 (ASGR1) for EVs produced by liver cells [38], microglial proteins (CD11b and CD45) for exosome-like vesicles found in cerebral spinal fluid (CSF) [39], the Clara cell protein 16 (CC16) for exosome-like vesicles present in bronchoalveolar lavage fluid (BALF) [40], ACE2 for SARS-CoV-2 spike protein-guided EV isolation from plasma [41] and the placental alkaline phosphatase (PLAP) for exosome-like vesicles produced by placental cells [42].

The size, membrane composition, and content of EVs are known to be heterogeneous and highly dependent on the cellular state, source, and the environmental conditions of the cells from which they originate [43]. As such, the composition of EV cargo (i.e., their miRNA, lncRNA, DNA, and proteins) has been shown to partially reflect the metabolic status of their cells of origin. Specifically, during periods of enhanced cellular stress and disease progression a high degree of cellular sorting into EVs is observed [44]. Although the content of EVs provides an indirect reflection of the state of the cell from which they are derived, it does not represent a mirror image of the cellular content, as EVs may be enriched in low-expressed RNAs or proteins from their cells of origin, while being depleted in others [45]. The molecular mechanisms involved in packaging their cargo are highly intricate and specialized processes [32]. The packaging of EV cargo, particularly in cancer cells, appears to favor the enrichment of specific proteins, lipids, and RNAs over others [46]. Proteins enriched within EVs derived from tumor cells have been shown to include heat shock proteins (HSP70, HSP90), export molecules (RAB27a/b, Alix, TSG101), proteases (ADAM10), and metabolic enzymes (enolase-α, glyceraldehyde 3 phosphate dehydrogenase, ATP synthase) [47]. EVs are also enriched with a variety of both short and long RNA species [48], including tRNA, vault RNA, and miRNAs [49].

The biogenesis and release of EVs is affected during disease, and in particular EV secretion from tumor cells is known to be exacerbated, which in turn enhances tumorigenesis and cancer progression [50]. The enhanced secretion and subsequent fusion of tumor-derived EVs with target cells, either locally within the TME or at a long distance in pre-metastatic tissues, facilitates the horizontal transfer of both their cell surface receptors and their internal cargos, which exhibit multifaceted and oftentimes double-edged functionality in recipient cells [51]. The complex composition of EV cargo during tumor progression coupled with their enhanced ability to travel through the body by diffusion, through the circulation, or within biofluids, further highlights that they are well adapted devices to establish and maintain the control of cancer cells over other normal cells. Additionally, because their cargo represents an encapsulated molecular snapshot of their cellular origin, they are potent reservoirs of biomarkers not only for initial cancer diagnosis but also for the monitoring of metastasis. Indeed, several studies have already demonstrated that EVs derived from tumor cells can play a significant role in promoting angiogenesis [52], stromal remodeling [53], enhanced immune surveillance [54], and most notably metastasis [55,56]. While the acidity of the tumor microenvironment is known to play a critical role in several cellular processes prominent in cancer, including chemotherapeutic resistance [57,58,59,60] and metastatic dissemination [61], several studies have suggested that the TME may itself contribute to the regulation of EV trafficking within the tumor mass [62]. For example, several studies have shown that the acidity of the tumor microenvironment contributes to the enhanced EV release and that it also facilitates the fusion of EVs with their recipient cells, through lipid–lipid interactions [62,63].

Considering the potent roles of extracellular vesicles and their local release in the tumor microenvironment, in this review we provide an extensive overview of EV trafficking within the tumor microenvironment, focusing primarily on the complex role that EVs play in orchestrating cancer-to-stromal cell communication within the tumor microenvironment and the pre-metastatic niche for the purpose of promoting the metastatic dissemination of cancer cells.

## 2. The Tumor Microenvironment

It is increasingly recognized that the complex heterogeneous nature of the tumor microenvironment plays a critical role in the evolution and progression of cancer. The communication between matrix cells within the TME, via extracellular vesicles, serves as an essential mediator for the development, recurrence, and metastatic dissemination of several cancer types [64]. The role of EVs within the tumor microenvironment appears to be multifaceted and bidirectional as the initial release of EVs from cancer cells leads to the recruitment and activation of stromal cells, which includes cancer-associated fibroblasts (CAFs), tumor-associated macrophages (TAM), cancer-associated endothelial cells (CAEC), and mesenchymal stem cells (MSC) [65]. As tumor growth progresses, evidence suggests that EVs released from surrounding stromal cells drive the epithelial-to-mesenchymal transition (EMT) of cancer cells, and thus their progression to more metastatic phenotypes [66,67]. Complementarily, as the release of EVs is enhanced during tumor growth, for certain tumors it allows their widespread diffusion and provides them with the potential to establish the pre-metastatic niche, which is necessary for the successful dissemination, colonization, and expansion of these cells to distant organ sites [68].

### 2.1. Mechanisms of Extracellular Vesicle-Mediated Metabolic Reprogramming and TME Remodeling in Metastasis

#### 2.1.1. Cancer-Associated Fibroblasts (CAFs)

Fibroblasts stem from a mesenchymal origin and are considered to be the most abundant cell type present in connective tissue (i.e., the supportive tissue of an organ) [69,70]. Through their secretion of collagen proteins, elastins, adhesive proteins (e.g., laminin and fibronectin), and ground substance (e.g., glycosaminoglycans), they maintain integrity of the extracellular matrix (ECM), thereby providing structural support to tissues and organs [71]. Under normal homeostatic conditions, fibroblasts remain quiescent as spindle-shaped single cells embedded within the ECM in the interstitial space, however, in response to tissue damage/injury they become reversibly “activated” to promote tissue repair during the wound healing process [72]. Once “activated” these fibroblasts display enhanced expression of α-smooth muscle actin (αSMA) and vimentin, and, together with increased ECM production and cytoskeleton remodeling, become stellate in shape (i.e., star-like) and gain contractile properties [6], at which point they are identified as being mesenchymal stem cell-like with characteristic signs of smooth muscle that has granted them the name of myofibroblasts.

In many instances, tumors are analogous to a wound as the expansion of new cells and chronic inflammation results in a continuous state of tissue injury, and the chronic activation of a wound healing/fibrotic response, and as such tumors are often referred to as wounds that do not heal [73,74,75]. Within the TME, fibroblasts that are “activated” by tumor cells are termed cancer-associated fibroblasts (CAFs), which typically constitute the most abundant stromal cell type present within the tumor microenvironment of solid tumors, including those of the colon, breast, and pancreas [76]. In pancreatic cancer for example, the tumor mass typically comprises 60–70% stromal CAFs, enhanced collagen, and other ECM components [77]. The intercellular communication between CAFs and tumor cells occurs via multiple ways, by direct cell–cell contact, by the transfer of secreted molecules, and by secreted extracellular vesicles that include EVs. It has been recently shown that the secretion of EVs is an important way for CAFs to influence the behavior of cancer cells (and vice versa) [78,79].

Understanding the role that EVs play in the communication between surrounding stromal cells and epithelial cancer cells, and vice versa, is critical for elucidating the influence of the tumor microenvironment during cancer cell progression and/or metastasis. The inherent mechanisms involved in the “activation” of CAFs within the tumor microenvironment appear to be related to EMT-induced differentiation of both resident stromal cells and recruited bone marrow-derived stem cells, which result in the formation of myofibroblasts. The initiation of CAF “activation” is predominantly driven by transforming growth factor beta 1 (TGF-β1) and fibroblast growth factor-2 (FGF-2) [80]. Interestingly, it has been recently shown that EV-derived TGF-β1 supplied by cancer cells is the only essential requirement for the differentiation of fibroblasts into CAFs [81], supporting the role of EVs for maintenance and modification of the tumor microenvironment. Similarly, ovarian and breast cancer-derived EVs have been shown to induce the conversion of adipose-derived mesenchymal stem cells into myofibroblast-like cells [82,83]. Within the TME, CAFs are predominantly responsible for the production of essential extracellular matrix proteins (such as fibronectin and collagens) and proteases [84]. It has been shown that increased production of these proteins leads to the progressive stiffening of the extracellular matrix, thereby facilitating tumor progression, vascularization, and metastasis [85]. The EV release of bioactive molecules, such as platelet-derived growth factor (PDGF), hepatocyte growth factor (HGF), interleukin-6 (IL6), proteases, and miRNAs [86] by CAFs into the extracellular matrix further supports their importance in cell–cell communication within the tumor microenvironment. In breast cancer for example, a study by Luga et al. showed that EVs released from surrounding stromal cells can promote breast cancer cell motility and metastasis through the mobilization of Wnt11-induced planar cell polarity [87]. Indeed, Luga and colleagues demonstrated that EVs released from cancer-associated fibroblasts (CAFs) promote the increased motility and metastatic capability of breast cancer cells, which is dependent on the interaction of breast cancer cell-produced Wnt11 and CAF-derived CD81 [87]. Another study on lung cancer cells also showed that microvesicles containing the extracellular matrix MMPs inducer (EMMPRIN) can stimulate the expression of matrix metalloproteinases (MMPs) in CAFs, which results in enhanced tumor metastization [88]. Other studies, performed on prostate cancer cell-derived EVs, which contain significantly increased levels of IL6, TGF-β, MMPs, carbonic anhydrase IX, and tumor necrosis factor 1α (TNF-1α) reported the induction of a stem cell-like phenotype thus enhancing metastasis under hypoxic conditions [89,90,91].

#### 2.1.2. Tumor-Associated Macrophages (TAMs)

Macrophages are a key population of innate immune cells responsible for executing a broad spectrum of functions ranging from the modulation of tissue homeostasis to the defense against pathogens and the facilitation of wound healing [91]. Macrophages that have infiltrated into the microenvironment of solid tumors are termed tumor-associated macrophages (TAMs) and form a critical component of the TME. Many of the TAMS located within the TME originate from bone marrow monocyte precursors and are recruited into the TME as a result of the tumor-derived chemoattractants that are continuously present within tumors. TAMs affect tumor growth, angiogenesis, metastasis, and chemoresistance. Within tumors, most TAMs gather at the leading edge and within hypoxic avascular areas [92], while a few also align along the abluminal side (i.e., away from the lumen) of the vessels [93]. They are recruited and activated by various signals in the TME and can have dramatic impacts on tumor progression and metastasis. TAMs have been demonstrated to perform a diverse range of immune regulatory functions and tumor progression, including that of promoting cancer cell proliferation and invasive capacity. In fact, tumor-elicited inflammation promotes tumor growth via the presence of the TAM-derived inflammatory cytokines interleukin (IL)-23 and IL-17 [94]. Moreover, an increase in TAM-derived IL-6 has been shown to contribute to STAT3 signaling induced hepatocellular carcinoma development and progression [95].

Macrophages can display different and even opposing phenotypes, depending on the microenvironment which they are embedded in. Once activated, macrophages are often classified as having either an M1 (classical-activated macrophages) or M2 (alternative-activated macrophages) phenotype [96]. M1 macrophages typically promote an inflammatory response against invading pathogens and tumor cells, whereas M2 macrophages tend to exert an immune-suppressive phenotype, which favors tissue repair and tumor progression. Each polarized macrophage type displays distinct expression profiles of genes, cytokines, and cell-surface markers [97]. Among those factors, colony-stimulating factor 1 (CSF-1) and C-C motif ligand 2 (CCL2) are the two most well-documented macrophage recruiters and M2-stimulating factors. CSF-1 is a potent determinant factor of macrophage polarization, as CSF-1 overexpression is often observed at the invasive edge of various tumors and correlates with a significant increase in metastasis [98].

Many studies have attempted to elucidate the crosstalk that exists between tumor and immune cells within the TME. Studies have shown that tumor-derived EVs play a vital role in the conversion of monocyte-derived macrophages into regulatory macrophages and in the mediation of cancer-related inflammation and tumor development [99,100] through the transfer of their cargos to recipient cells within the TME [101,102]. These cargos include proteins, nucleic acids, and lipids. Several studies have shown that depletion of EVs can disrupt the communication between tumor cells and TAMs, which reverses some of the harmful effects that EVs exert during tumor progression, restoring chemotherapeutic drug sensitivity [103,104,105].

In recent years, several studies have shown that EV miRNAs can play a crucial role in tumor progression, through their ability to regulate angiogenesis and facilitate metastasis by interfering with normal cellular programs of recipient cells [106,107]. Tumor-derived EV miRNAs have been shown to polarize recipient macrophages by targeting several signaling pathways, which can positively or negatively impact tumor progression [65,108]. It has been shown that tumor-derived EV miRNAs can promote cancer metastasis by regulating the crosstalk between cancer cells and TAMs, also providing a therapeutic strategy for cancer therapy. For example, colorectal-derived EVs carrying miR-203 are incorporated into monocytes [109], while EVs carrying miR-145 [110] and exosome-like vesicles containing miR-934 [108] are taken up by macrophages, which leads to their polarization into the M2 phenotype. M2 polarization induced by EVs derived from oral squamous cell carcinoma harboring miR-29a-3p has been shown to target (SOCS)1/STAT6 signaling, directly promoting tumor growth [111]. Whereas, hypoxia in ovarian cancer induces the production of EVs enriched in miR-940, miR-21-3p, miR-125b-5p, miR-181d-5p, and miR-222-3p stimulates macrophage M2 phenotype polarization and enhanced tumor growth [112,113]. Similarly, hypoxia-induced miR-301a-3p-enrichment in lung cancer-derived exosome-like vesicles results in a HIF1a/2a-dependent polarization of TAMs to the M2 phenotype, facilitating enhanced cell invasion, migration, and epithelial–mesenchymal transition (EMT) during lung metastasis [114].

#### 2.1.3. Tumor Endothelial Cells (TECs)

Angiogenesis, also known as new blood vessel formation, is essential for tumor progression and metastasis. The onset of angiogenesis occurs at any stage of tumor progression and depends on the type of tumor and its microenvironment. Endothelial cells, in the majority of solid tumors, are located within the inner layer of blood vessels and compared to normal endothelial cells have an altered morphology and molecular phenotype. Tumor blood vessels are characteristically unorganized, where they are often thin, fragile, and defective in barrier function resulting in leakiness of tumor blood vessels, whereas normal vasculature shows a hierarchal branching pattern of arteries, veins, and capillaries [115]. The unorganized nature of tumor blood vessels means that within specific focal regions they lack endothelial cells or basement membrane [116], which results in their exhibited chaotic blood flow (often termed leaky/hemorrhagic) [117]. Additionally, the high interstitial fluid pressure that is present within solid tumors causes blood vessels to collapse and further impedes blood flow. As such, hypoxic regions within the tumor tissue develop, despite the high level of vascularization [118]. During tumor formation and progression epithelial cancer cells actively secrete several pro-angiogenic factors which result in the excessive formation of abnormal blood vessels. Several studies have highlighted the differences between normal endothelial cells and tumor endothelial cells (TECs) [119]. In particular, the release of vascular endothelial growth factor (VEGF) in addition to other growth factors belonging to the ephrin and angiopoietin families from TECs is essential in promoting the formation of tumor blood vessels [120]. Hida et al. demonstrated that when compared to normal endothelial cells, TECs display several abnormalities [121,122] including differences in their responsiveness to epidermal growth factor (EGF) [123], adrenomedullin [124], and VEGF [125]. Ultimately, the differences in their response to these growth factors are important in the proangiogenic phenotype of TECs [122]. In particular, VEGF has been shown to stimulate cell migration and enhances survival of TECs in an autocrine manner.

**Figure 2:**
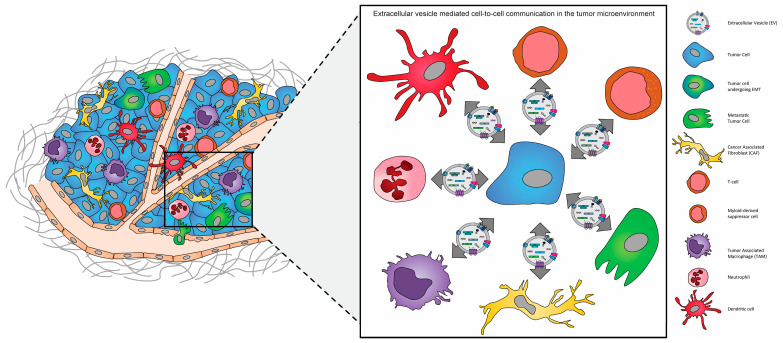
The “ecosystem” of the tumor microenvironment (TME). The tumor microenvironment of solid tumors is composed of extracellular matrix (ECM) components and a multitude of different stromal cells, including macrophages, dendritic cells, neutrophils, and myeloid-derived suppressor cells, adipocytes, fibroblasts, and endothelial cells, in addition to both non-cancerous and cancerous epithelial cells.

Several studies have demonstrated that like CAFs and TAMs, the infiltration and metabolic switch of endothelial cells to TECs within the tumor microenvironment supports and enhances angiogenesis during tumor growth and morphologically abnormal tumor vasculature promotes tumor cell intravasation during metastasis (Figure 2). VEGF–VEGF receptor signaling loosens the tight junctions that interconnect adjacent endothelial cells which renders blood vessels permeable to leakage. Additionally, high interstitial fluid pressures coupled with the immature structure of tumor blood vessels enhances the ease by which tumor cells permeate through tumor blood vessels [126]. Several researchers have demonstrated that TECs release specific growth factors, called angiocrine factors [127] into the TEM which convert indolent tumor cells to more aggressive cells displaying greater tumorigenicity, extranodal invasion, and chemoresistance [128,129]. Cao et al. showed that fibroblast growth factor 4 (FGF4), produced by B-cell lymphoma cells, activates fibroblast growth factor receptor 1 (FGFR1) in neighboring endothelial cells resulting in the upregulation of the Notch ligand Jagged1. In turn, Jagged1 on endothelial cells reciprocally induces Notch2–Hey1 signaling in lymphoma cells [128]. Mao and colleagues demonstrated that exosome-like vesicles derived from esophageal squamous cell carcinoma (ESCC) cells are mediators of intracellular communication between epithelial cancer cells and vascular endothelial cells within the TME. Specifically, they showed that hypoxic ESCC-derived EVs resulted in increased proliferation, improved capillary-like structure formation, and increased invasive ability of human umbilical endothelial cells (HUVEC), concluding that hypoxic EVs derived from cancer cells alter TECs within the TME, enhancing tumor angiogenesis [130]. To date, many studies have demonstrated that the miRNA, mRNA, and other non-coding RNA cargos of EVs released from tumor cells are responsible for the metabolic reprogramming of stromal cells, including TECs. For example, Chen et al. showed that exosome-like vesicles isolated from the serum of colorectal cancer (CRC) with metastases contain circTUBCGP4 which leads to miR-146b-3p inhibition in HUVEC cells, leading to Akt signaling pathway activation, which results in enhanced cell migration and angiogenic tube formation [131]. Biagoni et al. showed that urokinase plasminogen activator surface receptor (uPAR) containing exosome-like vesicles released from melanoma cells led to an increase in pro-angiogenesis of both human microvascular endothelial cells (HMVECs) and endothelial colony-forming cells (ECFCs), which they demonstrated was as a result of tumor exosome-like vesicle-mediated induction of vascular endothelial cadherin (VE-Cadherin), uPAR, and EGFR protein expression in endothelial cells [132]. In colorectal cancer cell-derived exosome-like vesicles, miR-25-3p has been shown to promote angiogenesis and the disruption of vein endothelial cell tight junctions within distant sites, including the lung and liver, helping to establish the pre-metastatic niche [133].

#### 2.1.4. Tumor-Infiltrating Lymphocytes (TILs)

Tumor-infiltrating lymphocytes (TILs) are defined as all lymphatic cell populations that invade into solid tumors. They consist primarily of cytotoxic CD8+ T cells and CD4+ helper T cells [134] in addition to smaller proportions of natural killer (NK) and B cells [135]. In all solid tumors TILs play distinct roles in modulating the TME, and for decades their role in tumor progression had been widely debated. In fact, the infiltration of immune cells into the TME and their role in cancer immunosurveillance is one of the hallmarks of cancer [136]. Although TILs have been shown to serve as somewhat of a double-edged sword as their infiltration into tumors can promote the initial establishment of a TME which is more susceptible to enhanced tumor progression [137], they can also attack tumor cells and in that way serve as potent tumor suppressors [138]. During the initial stages of tumor development, the infiltration of TILs and their prolonged interaction with surrounding tumor cells primes the hosts immune system against tumor cell elimination and in that way the tumor immunosurveillance results in the promotion of tumor growth [139]. However, in the long run the constant infiltration of TILs into the TME results in their exhaustion, characterized by sustained expression of inhibitory receptors distinct from functional effector and memory T cells, ultimately resulting in their failure to arrest tumor progression [138,140]. In fact, in a variety of tumor types (i.e., breast, colon, lung, and ovarian) the infiltration of immune cells into the TME has been shown to provide value as a predictive prognostic marker [141,142,143]. The treatment of metastatic cancer remains challenging, and in fact the study by Haj-Shomaly et al., 2022, suggests that paclitaxel chemotherapy, while effective in some cancer instances, may also promote tumor metastasis in the lung through its ability to rapidly induce ECM remodeling mediated by CD8+ T cells expressing lysyl oxidase (LOX), a potent ECM remodeling enzyme [144]. As such, in recent years tumor immunotherapy has become an attractive and effective treatment strategy for many solid tumor types. Several studies highlight the benefits of external expansion of TILs as an immunotherapeutic strategy, whereby TILs are harvested directly from tumor biopsies, expanded ex vivo, and then readministered to patients, referred to as adoptive cell therapy (ACT), with the main goal being to restore and enhance TIL anti-tumoral responses and the direct elimination of tumor cells [145,146,147],

Tumor-derived EVs are known to promote tumor progression through their direct modulation and suppression of the host immune response and chemoresistance and peripheral tolerance in cancer patients [148,149]. The cargo carried in tumor-derived exosome-like vesicles has been shown to include immunosuppressive molecules which influence the development, progression, and anti-tumor activity of immune cells either directly or indirectly [150]. Nakazawa et al., 2021, demonstrated that tumor-derived EVs containing CD300a are taken up by dendritic cells resulting in their inhibited secretion of interferon-β (IFN-β) leading to enhanced tumor immunity via the decreased activation of regulatory T cells [151]. Tumor-derived EV-dependent modulation of TIL activity occurs through the inhibition of proliferation and signaling activity of CD8+ T cells resulting in their apoptotic cell death [152]. Contrarily, dendritic cell-derived EVs promote the proliferation of T cells within the TME [153]. Cancer-derived CD8+ T cells from patients with head and neck cancer when co-cultured with tumor-derived EVs has been shown to induce the loss of CD27 expression in CD8+ T-cell and thus resulting in their change from the anti-tumor phenotype towards a more potent tumor suppressor phenotype [154,155,156]. Additionally, several studies have also suggested that tumor-derived EVs expressing the transmembrane protein FasL isolated from the plasma of oral cancer patients have the ability to induce apoptosis of CD8+ T cells [157,158]. Since regulatory T cells (Tregs) are critical for immune system suppression and the infiltration of Tregs into the TME, and their elevated presence in circulation is a strong prognostic marker in cancer [159], several studies have demonstrated that tumor-derived EVs promote the expansion of CD4/CD25/FOXP3 triple-positive Tregs and apoptotic induction of TILs [160]. Based on the immunoregulatory effects of EVs, which include the modulation of antigen presentation and immune activation and surveillance [161,162,163], multiple studies have investigated the ability of exosome-like vesicles to participate in tumor regression, demonstrating that immune cell-derived EVs display potent cytotoxic effects in hepatocellular carcinoma when administered as a cell-free anti-tumor vaccine [164,165].

Collectively, when considering the multifaceted and intricate role that EVs play in cell-to-cell communication between tumor cells, CAFs, TAMs, TECs, and TILs within the TME, it is no wonder that extensive research has been conducted to understand the mechanisms underlying EVs in tumor promotion and metastasis. Additionally, their enhanced secretion from tumors and the altered content of tumor-derived EVs when compared to EVs secreted from normal cells offer the potential to not only diagnose and monitor cancer progression, but also the generation of bioengineering EVs which interrupt the communication between tumor cells with the surrounding TME, thereby preventing EMT initiation, is an exciting and promising avenue in anti-metastatic therapy in cancer.

## 3. Extracellular Vesicle-Mediated Pre-Metastatic Niche Formation

The release of EVs from primary tumors selectively and favorably modifies the microenvironment of distant organs prior to the dissemination of metastatic cancer cells [166], termed pre-metastatic niche (PMN) formation. These changes to secondary organs, most commonly involving the lung followed by the liver, bone, and brain [167], have been shown to be caused by matrix metalloproteinase 9 (MMP-9), which is specifically induced in pre-metastatic lung endothelial cells and macrophages [168]. Hiratsuka et al. (2008) also demonstrated that in the lung, inflammatory PMN formation was promoted by the inflammatory mediator S100A8/A9 via a toll-like receptor (TLR)-dependent mechanism of induction of serum amyloid A (SAA) and the recruitment of Mac1+ myeloid cells [169]. Kaplan et al. (2005) demonstrated that the migration of VEGFR-1-expressing bone marrow-derived hematopoietic progenitor cells (BMDCs) into organ-specific pre-metastatic sites was induced by tumor-derived conditioned media and that BMDC cluster formation, which create a favorable microenvironment for incoming tumor cells, preceded tumor cell arrival [166]. Additionally, cell-free conditioned medium harvested from hypoxic breast cancer cells has been shown to drive the infiltration of CD11b+/Ly6Cmed/Ly6G+ myeloid-derived suppressor cells (MDSCs) and the reduction in cytotoxic natural killer (NK) cell populations, within the lung PMN of immune-competent mice [170]. In addition to the contribution of BMDCs to the generation of pre-metastatic niches, multiple studies have identified a large number of cytokines, chemokines, and growth factors which are connected to specific pre-metastatic niche-related processes [171,172,173]. In recent studies, EVs secreted from primary tumor cells have been described to have unique and central functions during PMN establishment and maintenance [174,175,176].

**Figure 3:**
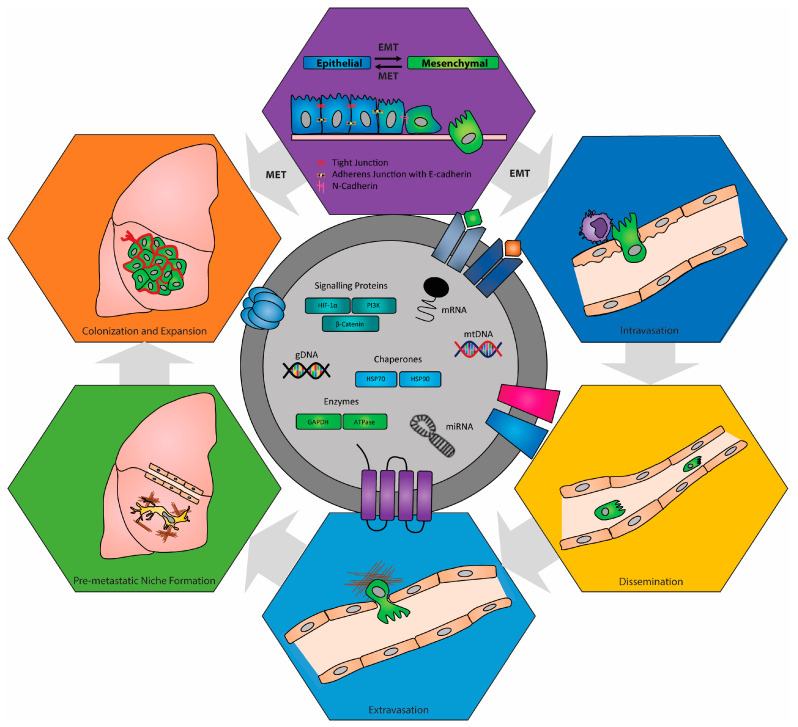
Tumor-derived EVs are critical mediators in tumor-cell metastasis. Extracellular secretion by tumor cells act as nanosized intracellular messages between cancer cells and surrounding stromal cells within the tumor microenvironment. Their cargo, which is composed of proteins, lipids, DNA, mRNAs, miRNAs, and other non-coding RNAs, is tailored by their cells of origin to specifically and directly promote cancer progression and metastasis by affecting epithelial-to-mesenchymal transition, tumor cell intravasation, dissemination, extravasation, and most critically, pre-metastatic niche formation.

Successful dissemination of tumor cells from primary tumor sites and the progression of metastasis is highly dependent on the establishment of a favorable pre-metastatic niche microenvironment through both the modulation of vascular permeability and the stimulation of neo-angiogenesis within secondary organ sites (Figure 3). It is precisely this modulation of endothelial activity that serves as one critical element of the pro-metastatic role that tumor-derived EVs play in the successful invasion and colonization of tumor cells during metastasis. Recently, the miRNAs contained within tumor-derived EVs have been extensively correlated with vascular remodeling and neo-angiogenesis. For example, miR-105 carried within EVs derived from the metastatic MDA-MB-231 breast cancer cell line increases metastasis by facilitating endothelial cell barrier destruction via the downregulation of the tight junction zonula occludens 1 (ZO-1) protein [177] and Golgi integral membrane protein 4 (GOLIM4) [178]. Hannafon et al. also demonstrated that EVs derived from the breast cancer cell lines MDA-MB-231, MCF-7, and BT20 displayed significantly different miRNA profiles when these cells were treated with anti-angiogenic docosahexaenoic acid (DHA) compared to those isolated from untreated cells [179]. They showed that the expression levels of 83 miRNAs were altered in MCF-7 cell-derived EVs following DHA treatment and that the EVs they isolated from DHA-treated MDA-MB-231 cancer cells contained abundant miRNAs associated with anti-angiogenesis, including let-7a, miR-21, miR-23b, miR27a/b, and miR-320b, which when taken up by endothelial cells reduced their angiogenic potential [179]. Indeed, miR-105 and miR-939 carried within EVs derived from metastatic breast cancer cells have been shown to increase vascular permeability, accelerating metastasis by facilitating colonization in new tissues [177,180]. Kosaka et al. demonstrated that exosome-like vesicles derived from breast cancer cells have also been shown to contain miR-210 which when taken up by endothelial cells induced a pro-angiogenic response and caused increased TEC migration and capillary-like network formation [181]. The importance of vascular remodeling of pre-metastatic organ tissue sites is supported by observations that EVs purified from CD105+ renal cancer stem cell supernatants enhance lung metastasis in vivo via the up-regulation of VEGF and MMP-2 in lung endothelial cells [182]. Collectively, these studies demonstrate that tumor-derived EVs strongly modify endothelial cell function, which contributes to the remodeling of both the primary tumor microenvironment and the PMN to promote tumor progression and metastatic dissemination. However, it is noteworthy to understand that although vascular remodeling is considered an essential step during the metastatic cascade, alone is not enough to promote tumor colonization and progression within secondary sites.

Cellular survival can be seen as the sole driving force of cancer cells; it is therefore not surprising that during cancer development and progression clonal selection is facilitated by the acquisition of beneficial mutations that downregulate tumor suppressors and/or upregulate oncogenes [183]. Tumor cells have therefore developed mechanisms to increase their resistance to chemotherapeutic drugs, which in the case of doxorubicin can occur through the upregulation of ATP-binding cassette (ABC) efflux pumps, enhanced nuclear export, continued topoisomerase IIa activity, and/or suppression of downstream apoptosis signaling [184]. Since EVs play critical roles in facilitating intercellular communication, cancer cells have been shown to package chemotherapeutic drugs within their cargo, and as such they actively participate in the increased resistance of cancer cells to chemotherapeutic treatment [185,186,187,188]. Zheng et al. (2017) demonstrated that the exosome-like vesicles that were isolated from M2 macrophages had the ability to confer cisplatin resistance to gastric cancer cells through the transfer of miR-21, which resulted in phosphatase and tensin homolog (PTEN) downregulation and the inhibition of PI3K/Akt signaling [189]. Ji et al. (2015) showed that in gastric cancer mesenchymal stem cell (MSC)-derived EVs induced fluorouracil resistance through the activation of the CaMK/Raf/MEK/ERK signaling pathway [190]. Additionally, several studies have also demonstrated that, in addition to the role that tumor-derived EVs play in establishing a suitable PMN, they also have distinct local effects within the primary TME wherein they promote the transformation of MSCs into tumor-like cells [191,192]. Boelens et al. (2014) demonstrated that stromal cells transfer EV-derived 5′-triphosphate RNA to breast cancer cells resulting in increased radiation resistance through the activation of retinoic acid-inducible gene I (RIG-I)-dependent NOTCH3 signaling [193].

Overall, considering the role that EVs play in the establishment of the PMN coupled with the fact a hospitable environment specifically tailored to the needs of each tumor cell type is critical for successful metastatic dissemination, the prospect of tailoring/targeting EVs to inhibit and prevent PMN formation in addition to the prevention of EMT initiation as described previously holds significant potential as a future gold-standard anticancer therapeutic avenue.

## 4. Concluding Remarks

EV studies that have individually investigated the different types of cells constituting primary tumors, as well as their interactions, have highlighted how tumor-derived EVs, their cargos, and their surface signaling molecules can directly reprogram their TME as well as the microenvironment of PMN to enhance tumor cell survival, proliferation, and particularly metastatic, invasive, and colonizing abilities. Furthermore, it is well established that tumor-derived EVs are also capable of modulating the extracellular matrix (ECM) in order to remodel both primary tumor sites, where metastasis can be successfully initiated, and generate secondary tumor sites, where they educate normal cells and establish metastatic niches. It is the precise and timely orchestration of these intricate signaling interactions between tumor-derived EVs and PMN cells that create hospitable environments, known as cellular education, which direct colonization and systemic expansion of cancer cells. Tumor-derived EVs have gained research interest in the quest to decipher the metastatic process, but they are very complex and independent cellular entities with unique multi-omic compound ratios, which uniquely orchestrate the cellular programs of tumor cells that maintain survival and direct expansion. Additionally, within the EV community it is widely accepted that tumor cells acquire an increase in output of EVs when compared to normal cells, and it is believed that this increase in tumor-derived EVs within the circulation is essential for successful PMN formation, metastatic dissemination, and secondary tumor expansion. This enhanced biological process has potential to provide detectable EV-based biomarkers for early diagnosis but also suggests that targeted therapeutic strategies aimed at reducing EV concentrations in the circulation may help control (reduce or redirect) metastatic processes in the human body. One may imagine the development of novel devices, which may help not only filter out metastatic EVs from the circulation by targeted selection, but also allow the circulation of EVs that may help reduce primary and secondary tumor growth in cancer patients.

## Data Availability

Not applicable.
